# Food Advertising to Children in New Zealand: A Critical Review of the Performance of a Self-Regulatory Complaints System Using a Public Health Law Framework

**DOI:** 10.3390/nu12051278

**Published:** 2020-04-30

**Authors:** Fiona Sing, Sally Mackay, Angela Culpin, Sally Hughes, Boyd Swinburn

**Affiliations:** 1 School of Population Health, University of Auckland, Auckland 1023, New Zealand; sally.mackay@auckland.ac.nz (S.M.); boyd.swinburn@auckland.ac.nz (B.S.); 2 Auckland Regional Public Health Service, Auckland 1051, New Zealand; aculpin@adhb.govt.nz (A.C.); sallyh@adhb.govt.nz (S.H.)

**Keywords:** advertising, self-regulation, children, food

## Abstract

New Zealand has the second highest overweight and obese child population in the Organisation for Economic Co-operation and Development (OECD). This paper evaluates whether New Zealand’s self-regulatory controls on the advertising of unhealthy food and beverages to children and young people adequately protects children from the exposure to, and power of, such marketing in order to limit its impact on children’s food and beverage preferences. First, an analysis of the relevant New Zealand Advertising Standards Authority (ASA) Codes was conducted, including the ASA Complaints Board and Appeals Board decisions from 2017–2019 to determine the application of the Codes in practice. Second, a public health law framework was applied to the self-regulatory system. Of the 16 complaints assessed, 12 were not upheld, and only one was upheld under the Children and Young People’s Advertising Code (CYPA Code). Three complaints were upheld under the Advertising Standards Code (ASC) but not the CYPA Code. An analysis of the Codes and their interpretation by the Complaints Board found that many facets of the public health law framework were not met. The self-regulatory system does not adequately protect children from the exposure to, and power of, unhealthy food and beverage marketing, and government-led, comprehensive, and enforceable marketing restrictions are required.

## 1. Introduction

Globally, rates of overweight, obesity, and diet-related non-communicable diseases (NCDs) are increasing [1]. Over 38.3 million children under 5 years of age are overweight, increasing lifetime exposure to the associated risks of obesity, including some cancers, cardiovascular disease, insulin resistance, musculoskeletal disorders, and disability [2]. Children with obesity are very likely to remain obese as adults and are at risk of developing serious NCDs [3]. New Zealand is rated as the second most overweight and obese country in the Organisation for Economic Co-operation and Development (OECD), with 40% of children aged 4–19 years old overweight or obese [4]. Transnational corporations produce high fat, sugar, and salt (HFSS) products and engage in influential marketing techniques, which influence children’s eating behaviors, preferences, requests, nutrition knowledge, and food intake [5,6,7,8,9,10,11]. Evidence shows how marketing of these foods is linked to unhealthy weight outcomes [11]. International consensus, including the WHO Commission on Ending Childhood Obesity, calls for comprehensive marketing restrictions to be implemented to reduce the effect of advertising of unhealthy products on dietary behavior and the related health outcomes [3,9,12,13,14,15,16]. Many governments have voluntary systems, including industry self-regulatory systems, to regulate the marketing of unhealthy food to children [17,18,19]. In New Zealand, the Advertising Standards Authority (ASA), an industry-run organisation with 14 membership organisations representing advertisers, agencies, and the media and an industry-run governance board, self-regulates advertising funded by member subscriptions and advertiser levies.

Independent evaluations of effectiveness of both government-led voluntary regulation and industry-led self-regulation indicate that the impact of both approaches on reducing the exposure and power of marketing to children is limited [20,21,22,23,24,25,26,27,28,29,30,31,32,33,34]. Impact studies in Australia found that the frequency of food advertising and children’s exposure to unhealthy food marketing remained unchanged despite the implementation of industry self-regulatory pledges [22]. Similar results were found in Canada, Germany, Spain, and the US [20,25,26,27,33,35,36]. Research in New Zealand found that the majority of foods advertised in peak children’s TV viewing times were classified as those not permitted to be advertised to children under the WHO nutrient profile model [23]. This is in contrast to evidence from Chile that shows that a comprehensive mandatory marketing restriction can reduce the exposure of unhealthy food marketing to children, with significant decreases found in exposure to advertising on TV and child-directed marketing strategies on breakfast cereal packages post-implementation of the statutory marketing regulation [37,38]. Such impact studies focus on evaluating outcome measures, such as the frequency and/or volume of advertising during children’s peak viewing times and the nutritional quality of foods and beverages promoted to children. What is not commonly explored is how these regulatory systems are performing in practice to understand how they are failing to reduce the frequency and volume of advertising or improve the nutritional quality of foods and beverages promoted to children.

The paper seeks to complement the impact studies by analysing the quality of the existing regulatory regime in New Zealand using a public health law framework. The objective of this approach is to interrogate the design, administration, and enforcement of the regulatory system to ascertain whether the approach is effectively improving the food marketing environment in New Zealand and, if not, exactly how the approach is failing. The ineffectiveness of a regulatory system to adequately reduce the exposure of unhealthy food marketing to children has knock on effects on dietary behavior and health outcomes, such as overweight and obesity.

## 2. Methods

A two-staged assessment took place. First, we examined both the relevant Codes and the complaints made to the Complaints Board and its corresponding decisions to understand the interpretation and application of the Codes. Given the vague language of the Codes, the additional analysis of the complaints gave a more accurate evaluation of the regulatory system than an analysis of the substantive content of the Codes alone. In the second stage of the assessment, a public health law framework developed by Reeve and Magnusson [39] was applied to evaluate whether New Zealand’s self-regulatory approach exhibits the features of an effective, transparent, and accountable regulatory regime that adequately protects children from exposure to unhealthy food marketing. We chose to focus on the Children and Young People’s Advertising Code (CYPA Code), since one of its main aims is to protect children from unhealthy food and beverage marketing. However, as multiple complainants used both the CYPA Code in tangent with the Advertising Standards Code (ASC), we broadened our scope to assess the ASC as well. The CYPA Code states that it applies to all advertisements that target children or young people “whether contained in children’s or young people’s media or otherwise”. The ASC is also relevant to food and beverage advertising as it governs responsible advertisements and complaints have been made regarding unhealthy food advertising under the ASC.

The selection criteria for the complaints were that the complaint had to be brought under the CYPA Code, or both the CYPA Code and the ASC, and the complaint had to relate to advertising to children of HFSS foods defined as occasional foods under the New Zealand Food and Beverage Classification System (FBCS system). The CYPA Code came into force in 2017, and therefore the time period reviewed was complaints made between 2017–2019.

The public health law framework by Reeve and Magnusson evaluates regulatory controls on the marketing of food and beverages to children to identify strengths and weaknesses of regulatory models and whether they effectively improve the food marketing environment [39].

The framework comprises three domains:

1. The substantive content of regulatory standards:

- Regulatory purpose;
- Substantive terms and conditions.

2. The design of regulatory processes for the administration of the regulatory scheme:

- Drafting regulatory rules and scheme design;
- Administration and monitoring;
- Review.

3. The enforcement of standards:

- Complaints handling;
- Enforcement.

For each element within the domains of the Reeve and Magnusson framework, a benchmark of best practice is set for an effective, transparent, and accountable regulatory regime that would adequately reduce the exposure of children to unhealthy food marketing. The ASA system (the Codes and the Complaints Board decisions) was assessed against this benchmark to assess how the system performed compared to the best practice standard.

## 3. Results

### 3.1. Summary of Decisions

Sixteen complaints and two appeals met the criteria for analysis between 2017 and 2019. Eight were brought under the CYPA Code solely, six under both the CYPA Code and the ASC, and two under the CYPA Code and other Codes (Advertising Code of Ethics and Code for Advertising Food (the predecessor to the ASC)). Table 1 provides a summary of the complaints and decisions. The advertising mediums included a mix of television advertisements, online advertisements (Facebook, websites, Instagram), outdoor advertisements (outside shops, street signage, bus shelters—otherwise known as ’out of home’ advertisements), and print advertisements. The marketing techniques used included using famous sports stars, children, digital techniques, such as emojis or characters like Santa Claus, and advertising limited edition offers, major food and beverage industry-sponsored events, or advertising the ‘healthful’ nature of the products (i.e., a healthy burger or pizza options). Other advertisements suggested ways children could consume products, such as cookies for breakfast cereal, chocolate eggs ‘made for kids’, or biscuit packets in lunch boxes. Radio is an uncommon medium for advertising to children and there were no complaints about advertisements on radio.

Two complaints were upheld under the CYPA Code; however, one was appealed and the decision reversed. Four complaints were upheld under the ASC; however, one was appealed and the decision reversed. Two further complaints were “settled”, and one complaint was partially settled, meaning that the advertiser removed the advertisement before the Complaints Board adjudicated on the complaint, therefore no decision was given. Thus, of the 16 complaints under the CYPA Code, 12 were not upheld, and only one was fully upheld.

The analysis of how the Codes were interpreted by the Complaints Board to reach the final decisions provides additional context about how the regulatory system performs in practice.

### 3.2. Application of Public Health Law Framework to the ASA System

#### 3.2.1. Evaluating the Substantive Terms and Conditions of the Regulatory System

##### Regulatory Purpose: CYPA Code

An effective marketing regulation should have a stated objective of reducing children’s exposure to, and the persuasive power of, marketing of unhealthy products to provide an important standard to measure the performance of the system [3,13,39]. Regulatory systems are weaker if they have vague objectives that are not aligned with reducing exposure and power of marketing practices [39].

The stated purpose of the CYPA Code is to recognise the need to protect children under the United Nations Convention on the Rights of the Child (UNCROC); it is a commendable objective; however, there is no mention of reducing exposure or power of marketing. In order to fulfil the UNCROC, a strong regulatory system that effectively reduces the exposure of children to harmful marketing practices, is required [20]. This analysis of complaints demonstrates that the ASA system is not fulfilling that objective.

##### Substantive Terms and Conditions: CYPA Code

A robust regulatory system that effectively restricts exposure and power of marketing to children would address the following key components [13,19,39]:

- Accurately capture the full extent of advertising that children are exposed to in the scope of the regulation;
- Use a definition of children that protects children up to 18 years;
- Capture the full range of mediums and techniques used to advertise to children and
- Use a comprehensive nutrient profiling system to define which food and beverages are subject to the regulatory system.

##### Accurately Capture the Full Extent of Advertising that Children are Exposed to in the Scope of the Regulation

The CYPA Code applies to all advertisements that target children, whether contained in children’s or young people’s media or otherwise. Of the 16 complaints adjudicated on by the Complaints Board under the CYPA Code, only one advertisement was deemed to be “targeting children”. For an advertisement to be classified as “targeting children or young people”, the context of the advertisement needs to be analysed, as well as the relationship between the following three criteria:

1. Nature and intended purpose of the product or service being promoted is principally or generally appealing to children or young people;
2. Presentation of the advertisement content (e.g., theme, images, colors, wording, music, and language used) is appealing to children or young people;
3. Expected average audience at the time or place the advertisement appears includes a significant proportion of children or young people.

In practice, the Complaints Board requires all three criteria to be met, and even where the product and presentation of a product is appealing to children or young people, the Complaints Board considers the harm can be mitigated by the placement of the advertisement.

The most common ground given by the Complaints Board for not upholding a complaint is that the expected average audience of the advertisement would not include a “significant proportion of children or young people”, which is deemed to be 25% or more of the expected audience. This definition was derived from advertisements on television, but the flaw of having adult viewership included in the denominator means that more adults in the audience will reduce the percentage of children watching but not the total number of children watching. Therefore, the restrictions may not limit children’s actual exposure to unhealthy food marketing, leaving unprotected the many children who watch mixed-audience programming or online activity and outdoor advertising [9,12,14].

The Complaints Board did not uphold certain complaints claiming that the intended audience for the advertisements were parents not children. An appeal decision (Appeal Decision 1) found that an advertisement with a school boy promoting a small biscuit packet of teddy bear biscuits in a child’s lunchbox advertised in bus shelters was targeting adult shoppers and not children because the nature and intent of the product and the presentation of the product was aimed at parent shoppers not children.

##### The Definition of Children

The Reeve and Magnusson framework defines a robust regulatory system as one that protects children up to 18 years old, in line with the UNCROC and other international recommendations [13,14,39].

While the CYPA Code does include young people aged 14 and up to 18 years old in its scope, it affords the age group different protections. While Rule 1 (i) states that advertisements of occasional foods *must not* target children, Rule 1 (j) states that young people can be targeted, but a “special duty of care” must be applied, a vague and less stringent rule. The guidelines state that advertisements must not state or imply that such products are suitable for frequent or daily consumption and, where possible, healthy or better-for-you options should be promoted. Other guidelines state that, where occasional food and beverages are advertised to young people, they should not be portrayed in any way that suggests they are beneficial to health. While sponsorship advertisements must not target children, “a special duty of care” must be taken for young people and must not show an occasional food or beverage product, such a product’s packaging, or depict the consumption of an occasional product.

None of the complaints made regarding young people have been upheld by the Complaints Board and the interpretation of a “special duty of care” has not been articulated expressly by the Complaints Board beyond accepting that advertisers put age limits on who sees the advertising campaign online (for example, Complaint Decision 11).

##### Capture the Full range of Mediums and Techniques Used to Advertise to Children

The CYPA Code defines “Advertising and Advertisements” as “any message, the content of which is controlled directly or indirectly by the advertiser, expressed in any language, and communicated in any medium with the intent to influence the choice, opinion, or behavior of those to whom it is addressed”.

While the definition of advertising is broad, when analysing the Complaints Board’s decisions, it is apparent that the scope of the advertising mediums and techniques that are restricted by the self-regulatory system is limited. Various advertising mediums and techniques have been the subject of the complaints made to date, but only one complaint, regarding outdoor advertising using Santa Claus (Complaint Decision 4), has been upheld under the CYPA Code.

In nearly all the decisions, marketing techniques, such as the use of famous sports stars, popular children’s characters, such as Santa Claus, the use of other children in the advertisements, limited edition offers, and the use of emojis, were considered to be appealing to children. However, the mediums they were communicated on that included TV advertising, online advertising (websites, Instagram, and Facebook), and outdoor advertisements were judged not to meet the criteria for targeting children or young people. The narrow interpretation of “targeting children” or “targeting young people” reduces the scope of the mediums and techniques covered by the CYPA Code.

##### Use a Comprehensive Nutrient Profiling System to Define Which Food and Beverages are Subject to the Regulatory System

The CYPA Code uses the New Zealand FBCS to determine which food and beverages are considered occasional and therefore subject to the restrictions. However, this classification system was not designed for advertising standards, but rather for classifying the type and portion size of food and beverages that should be sold in New Zealand schools. In addition, the FBCS has been retired by the Ministry of Health of New Zealand, and a new classification system for education settings is under development. This means the FBCS will no longer be a Government endorsed system, making it obsolete [40].

The WHO Regional Offices have designed nutrient profile models that are specifically intended for governments to use when restricting marketing to children, which would be more robust and appropriate classification systems to use, but are not used in the ASA system [41,42,43]. Under these models, foods are unable to be marketed to children if they include nutrients of concern that exceed the prescribed threshold based on per 100g only, not per serve. Under the WHO Western Pacific Regional Model, any form of cakes or biscuits are prohibited from being marketed to children, regardless of their nutritional composition, i.e., no thresholds are provided [43].

The FBCS system uses both a per 100g ratio and a per serve ratio as its thresholds, depending on the product category. For example, a small packet of children’s mini biscuits could be considered a “sometimes” food rather than an “occasional” food because the quantity of the product is under the prescribed serving size threshold. Using a serving size metric for products such as “sweet snacks” (biscuits, cakes, pastries) that are high in fat, sugar, or salt is inadequate, as the nutritional composition of the product should be the chosen metric of whether the food can be marketed to children not the quantity in the serving size. Such products should be prohibited from being marketed to children, as is the case in the WHO nutrient profile models.

In a comparison study conducted to ascertain which nutrient profiling systems to classify foods would best protect New Zealand children from exposure to the marketing of unhealthy food and beverages, it was found that the WHO Regional Office of Europe model would permit marketing of 29% of the products in scope, while the FBCS system would permit 39% of products. The FBCS system was found to permit marketing of a number of food products of concern, particularly high-sugar breakfast cereals, fruit juices, and ready meals [44].

##### Regulatory Purpose: ASC

The ASC states as its purpose that all advertising must be legal, decent, honest, and truthful and respect the principles of fair competition, so that the public can have confidence in advertising. This is a general regulatory purpose that does not specifically mention reducing exposure or power of advertising to children but is in line with the general purpose of the ASC.

##### Substantive Terms and Conditions: ASC

The ASC does not have comparable terms and conditions in relation to the components of targeting children with advertising. However, given that more complaints have been upheld under the ASC and not the CYPA Code, an exploration of the substantive terms and conditions and the ASA’s interpretation of the ASC is important.

Of the relevant complaints made under the ASC (n = 6), all contained a complaint under Principle 1 (Advertisements must be prepared and placed with a due sense of social responsibility to consumers and to society), in particular, Rule 1(h) (Advertisements must not undermine the health and well-being of individuals). Two complaints were also made under Rule 2(g), which states that Food and Beverage claims must be factual and able to be substantiated and must not be misleading.

The ASC provisions protect the general public: Children or young people are not uniquely protected and, as such, the advertising in question does not need to meet the same “targeting” criteria as the CYPA Code. The definition of advertising is the same as the CYPA Code and “foods high in salt, fat, and sugar” are the subject of the provisions, with no thresholds provided for what is considered “high in”.

The Complaints Board upheld four complaint under Rule 1(h) finding that the advertisements in question were socially irresponsible or undermined health and wellbeing (or both) (Complaint Decisions 8–10 and 16). The complaints that were upheld consisted of a mixed range of mediums such as bus shelter advertisements, Instagram, or website advertisements, and usually involved children in the advertisements. One complaint was upheld under Rule 2(g) as the advertisement was found to give false information about the nutritional value of the food product in question (Complaint Decision 10).

Multiple complaints were upheld under the ASC but not under the CYPA Code (Complaint Decisions 8–10 and 16). For example, the biscuit advertisement in a child’s lunchbox advertised at a bus shelter was determined to be socially irresponsible and undermining health and wellbeing, whereas it was not considered to be “targeting children” under the CYPA Code (Complaint Decision 9) [45].

#### 3.2.2. Evaluating the Regulatory Process Governing the Regulatory System

##### Rule Development and Scheme Design

The public health law framework stipulates that regulatory processes governing a regulatory system should include transparency and accountability mechanisms from their commencement, including when developing substantive regulatory rules and in determining scheme design. For example, working groups consisting of government agencies, public health organisations, and consumer advocates can be involved in the drafting of the regulations or external stakeholders could be consulted with [39].

As is typical for a self-regulatory system, the ASA Codes and Complaints system was designed by the ASA, which is made up of advertising industry stakeholders, but an external public advisory board consisting of different stakeholders, including public health, did advise the process.

The ASA did consult the public on the amendment of the CYPA Code and received submissions from various stakeholders, including public health stakeholders, on how the system could be improved. Some of the recommendations within the submissions were incorporated, but more substantial recommendations were not [42].

##### Administration and Monitoring of Performance and Compliance and Independent Review of Restrictions

The public health law framework states that an independent body should administer the regulatory system including monitoring and enforcing the system [39]. The independent body can organise a regular and structured external review of the system’s performance against its objectives [39]. The review should include baseline and follow-up data and timeframes for evaluation and should be conducted by independent third parties [39].

As is typical of a self-regulatory system, the ASA administers the New Zealand regulatory system. It is not an independent body with no conflicts of interest. The ASA states that the Codes will be reviewed every 5 years, or earlier if the need arises, and the CYPA Code is due for review in 2022. It is unclear what a review entails, but there is no provision in the ASA governing rules for an external review of the performance of the system by an arm’s length party, nor collection of baseline data, outcome indicators, stakeholder’s compliance with the Codes beyond the complaints process, or the scheme’s success in meeting its stated objectives.

#### 3.2.3. Evaluating Enforcement of Regulatory Standards for Food Advertising

##### Complaints Handling Mechanisms

The public health law framework states a complaint-handling scheme should be independent and credible so that the public feel confident using it and so that any amendments can be made to improve the system. Publishing the decisions on each complaint ensures the system is transparent and that a series of precedents can be developed to help understand the regulatory schemes’ terms and conditions [39].

The ASA complaints system is self-managed by the ASA through a Complaints Board and is therefore not independent. The Complaints Board is made up of a Chairperson, four public members, and four industry members. While the public members have no connection with the media or advertising industry, the ASA appoints the members.

The Complaints Board adjudicate on each complaint, including the advertiser’s response to the complaint, and publish the decision on the ASA website. The complainant and the advertiser can appeal the decision to the Appeals Board, who also publish the final decision online. Evidence shows that parents are either unaware of the ASA system or feel they are powerless in the complaints process, relative to the food industry [46]. Filing a complaint takes skill and time, which creates barriers for participation in the process. A complainant must include the time, date, and channel information when the advertisement was viewed, which the general public may not have access to, whereas the advertiser will hold this information. The complainant must also ascertain whether the product in question meets the nutritional criteria of “occasional” food under the FBCS, including the per serve metric for certain unhealthy food product categories.

In some cases, advertisers voluntarily removed the advertisement before a decision was issued, effectively “settling” the case. For example, Frucor removed an advertisement before the Complaints Board could adjudicate on the case, resulting in no decision being given (Complaint decision 1) [47]. This meant that the opportunity of developing a precedent for the Complaints Board to use in later decisions and for other advertising practices to follow was lost. This practice also has the effect of taking removed advertisements outside of the scope of the Codes, even when they have breached the Codes.

##### Enforcement

The public health law framework states that a range of enforcement mechanisms including incentives to encourage compliance, less strict measures like persuasion and more strict measures such as fines should be utilised to increase compliance [39].

The ASA system relies on the public reporting breaches of the ASA system, placing the onus of monitoring and enforcing the Codes on the general public, not the industry being regulated or an independent governing body.

If a complaint is upheld under the CYPA Code, the sole penalty is to remove the offending advertisement. The Complaints Board only considers the advertisement seen by the complainant, for example, one advertisement at one bus stop, instead of applying the decision to the whole advertising campaign. As a complaint regarding a broadcast advertisement has not been upheld, it is unclear whether an advertisement at a certain time period would be removed, but the whole campaign could continue on broadcast. Under the ASC, an advertiser is required to remove the offending advertising campaign across the whole medium (i.e., Instagram), but not the whole mixed media campaign if other elements of the campaign were found not to be in breach or were not raised by the complainant. This results in the wider advertising campaign continuing to impact children and provides very little incentive to advertisers to adhere to the Codes.

The decisions of the Complaints Board are sent to the media and posted on the news section of the ASA website. There is no financial repercussion or a limited name and shame element to a decision being upheld by both common enforcement mechanisms in other similar systems [48].

## 4. Discussion

This paper seeks to complement studies measuring the impact of marketing regulations on children by analysing the quality of the existing regulatory regime in New Zealand using a public health law framework. Applying the public health law framework to the ASA self-regulatory system (the Codes and the Complaints Boards decisions) allows for an assessment of whether the system is adequately regulating the food marketing environment in order to limit the impact of marketing on children’s food and beverage preferences.

The strengths of this paper are that it provides a different method to understand why a self-regulatory system is ineffective at reducing the power of, and exposure to, unhealthy food marketing to children. Qualitative approaches can provide context to explain how systems are operating and identify the strengths and weaknesses, giving more indication of how such regulatory systems can be improved. The limitations of this study are that there are relatively few complaints to assess and a bigger sample size may provide more of a robust insight into the regulatory system. A further limitation is that, where an advertisement was removed before a complaint was settled, no decision was given, reducing the sample size further and the jurisprudence available to analyse.

The results of this research provide evidence of why self-regulatory approaches are often found to have little effect in the various impact studies [20,22,24,25,26,27,33]. This includes research in New Zealand measuring unhealthy food advertising on broadcast television that found that the majority of foods (88%) advertised in peak children’s viewing times were classified as those not permitted to be advertised to children under the WHO nutrient profile model [23].

Reeve and Magnusson applied the public health framework to six jurisdictions, the United Kingdom, Australia, Ireland, Canada, Quebec, and the United States, which represented three different forms of regulation (self-regulation, co-regulation, and statutory regulation). They found that all jurisdictions failed to adequately protect children from exposure to marketing of unhealthy food, largely because of significant loopholes in the substantive provisions of the regulatory instruments, as well as limitations in the monitoring, review, and enforcement of the regulations [37]. Evidence from Chile confirms that a comprehensive mandatory marketing restriction can reduce the exposure of unhealthy food marketing to children and that further restrictions planned as part of the staged implementation of the law will further decrease the exposure of children to unhealthy food marketing [37,38].

In analysing the Codes and the interpretation of the Codes by the Complaints Board, it is apparent that many facets of the Reeve and Magnusson framework are not met. In relation to the substantive content of the government-led regulatory system, on reading the Codes, particularly the CYPA Code, the objective and provisions appear to be comprehensive. Protecting children under the UNCROC is commendable, the definition of advertising is broad, and children up to 18 are protected; however, reducing the power and exposure of marketing to children is not expressly mentioned. In reality, the definition of “targeting children” reduces the scope of the mediums and techniques that are covered by the CYPA Code, young people are not afforded the same protection as children, and vague definitions are included for key attributes of the system, for example “a special duty of care”. In addition, the FBCS classification system is too lenient and does not preclude enough unhealthy food products from being advertised to children.

Only one of the 16 complaints in scope was upheld by the Complaints Board under the CYPA Code; a Code introduced specifically to protect children. In comparison, the broader ASC has more successfully protected children from advertising of unhealthy food and beverage advertising, as the provisions of the ASC are less stringent and more able to protect children from socially irresponsible advertising that may undermine their health and wellbeing. The percentage of the complaints about occasional food and beverage advertising that have been upheld under the ASC in comparison to the CYPA Code calls into question whether the CYPA Code is achieving its aim of protecting children. The provisions of the ASC appear more robust in relation to protecting children from advertising, despite the CYPA Code being implemented with that intended purpose.

The Complaints Board’s interpretation of the definition of “targeting children” under the CYPA Code indicates that it does not regard basic marketing principles that use techniques such as the ‘pester power’ of parents and adopts a simplistic view that as children do not have purchasing power they are not the subject of advertisements of consumable products. Furthermore, the regulatory system has little capacity to deal with the changing pace of integrated mixed multi-media campaigns and the cumulative effect those campaigns have, which indicates that the current system is not adequate. Widening the interpretation of the Codes to address a whole advertising campaign would not only more effectively reduce the exposure and power of the advertisement to children, it would also provide a stronger incentive to advertisers to comply with the Codes to avoid any impact on the investment into a full media campaign.

The lack of an independent body or external stakeholders administering the regime and measuring the performance of the regime creates governance issues and impacts the ability of such a system to effectively reduce the power and exposure to children. Conflicts of interest plague self-regulatory systems, as industry stakeholders have conflicting mandates and ultimately their purpose and fiduciary duty is to protect their profits and shareholder value, not to protect children’s rights or public health.

The onus is placed unfairly on the consumer to uphold the system, and the evidence-base needed to effectively defend the complaint is biased towards the industry stakeholders. The results of the application of the public health law framework highlight the weaknesses with the system and call into question the effectiveness of the ASA to meet its own objectives, but also to effectively reduce the exposure and power of marketing of unhealthy food to children.

A self-regulatory system will inherently fall short of the public health framework set out by Reeve and Magnusson. To effectively reduce the exposure and power of harmful marketing practices of unhealthy food and beverages to children, an overhaul of the regulatory system is required. Firstly, a mandatory government-led system is needed, which allows for a governance structure that increases transparency and accountability. Government can set the substantive content of the regulatory system, including a monitoring and compliance system, and allow for independent review periods of the system to measure its performance against its overall objectives.

Older children must be afforded the same level of protection as younger children in line the with the UNCROC. The definition of marketing should be broad to encapsulate the range of media that children are actually exposed to [14]. Thresholds that focus on the proportion of children compared to adults as the indication of the audience base will not accurately capture the level of children in the audience. Using children’s viewership is a more logical and robust way to define marketing to children than including adult viewership in the metric. For example, in Quebec, the Consumer Protection Act defines advertising as targeting children in a similar way but removes the adult viewership metric. The Act defines advertising targeting children based on a) the nature and intended purpose of the goods advertised; b) the manner of presenting such advertisement; and c) the time and place it is shown [42]. Using a nutrient profile model that was developed for the purpose of classifying foods and beverages that should not be marketed to children will ensure that the correct scope of products is brought within the regulations.

Government monitoring of the marketing practices of industry stakeholders would remove the onus from the public to uphold the system and more comprehensive regulatory powers could be implemented that compel industry to divulge their advertising data. Stronger enforcement mechanisms, such as fines, naming and shaming, and partial bans on advertising, would provide more of an incentive for industry stakeholders to comply with the regulations. The cumulative effect of the entire marketing campaign should be measured and subject to the penalty. In Chile, enforcement provisions include penalties for violation, reprimands, fines, and prohibition from selling the advertised product, and, in the UK, persistent violators can be fined or have their broadcasting license withdrawn for non-compliance with broadcast regulations [48].

Governments have been called to enact comprehensive mandatory marketing restrictions by multiple international institutions, including the United Nations Committee on the Rights of the Child [49,50,51,52]. As a signatory to the UNCROC, the New Zealand Government, not industry, has a legal obligation to protect the rights of children, including their right to the highest attainable standard of health [51,52]. Globally, voluntary systems, including self-regulatory systems, are more common than mandatory systems [20]. The food and beverage industry prefer to self-regulate to avoid government interference in their marketing practices and claim that such systems are effective, despite evidence to the contrary [20]. The New Zealand Government has not regulated the marketing practices of the food and beverage industry, instead calling on the industry to suggest ways to improve the food environment [45]. This analysis of the system shows that a claim by the food and beverage industry that the New Zealand self-regulatory system is sufficient to reduce the adverse health outcomes, caused by the exposure to and power of harmful marketing practices, is unfounded and a distraction tactic from the implementation of the government-led mandatory regulatory approach required.

## 5. Conclusions

An application of the public health law framework to the New Zealand regulatory system confirms that the current self-regulatory system is ineffective at reducing the exposure to, and power of, marketing to children. This paper adds to the research by interrogating the interpretation of a self-regulatory code by a self-regulated Complaints Board through an analysis of its jurisprudence to understand how the relevant Codes are working in practice.

The results found that the self-regulatory system is not an effective, transparent, or accountable regulatory regime and that government-led, comprehensive, and enforceable marketing restrictions are required to effectively reduce the exposure to, and power of, marketing to children. Given New Zealand’s unacceptably high rate of childhood overweight and obesity, adequately regulating the food marketing environment is imperative in order to limit the impact of marketing on food and beverage preferences and the related health outcomes, such as overweight and obesity. Stronger marketing restrictions will also ensure that New Zealand fulfils its legal obligation to uphold the UNCROC. An update of the regulatory objectives, the substantive terms and conditions, and the governance structures around administering, monitoring, and enforcing the system by an independent body that does not have conflicts of interest is required to reduce children’s exposure to unhealthy food and beverage advertising.

## Figures and Tables

**Table 1:** Summary of complaints.

Complaints	Grounds for Complaint	Medium/Technique	Decision
**1. Frucor, Pepsi Max, Out of Home (12/09/17)**	CYPA—Principles 1; Rule 1(a), 1(g), 1(h), 1(i), 1(j), 1(l), Principle 3, Rule 3(a), 3(b)	Bus shelter Limited edition product with emojis and famous sports people	**Settled** as ad removed
**2. Frucor, Pepsi Max, Facebook (12/09/17)**	ASC—Rule 1(a), (g), (h), Rule 3 (a) and (b) CYPA—Principle 1, Rule 1(a), 1(g), 1(h), Principle 3, Rule 3(a), 3(b)	Online advertisement—Facebook Limited edition product with emojis and famous sports people	**Not Upheld**—ASC Rule 1(a), (g), (h), Rule 3 (a) and (b) CYPA—Principle 1, Rule 1(a), 1(g), 1(h), Principle 3, Rule 3(a), 3(b)
**3. Coca-Cola Oceania (13/02/18) Digital and Print media**	CYPA—Principle 1 Rule 1(a), 1(i), 1(j), Principle 2, Principle 3, Rule 3(a), 3(b) Advertising Code of Ethics—basic Principle 4	Digital, billboard, bus shelter and print advertisements of Coca Cola sponsored Christmas event using Santa Claus. Sponsored Event itself	**Not upheld**—CYPA Principle 1—Rule 1(a), 1(i), 1(j), Principle 2, Principle 3, Rule 3(a), 3(b) Advertising Code of Ethics—basic Principle 4
**4. Coca Cola Oceania, Out of Home, Poster, (13/02/18) 17/454**	CYPA—Principle 1, Rule 1(h), 1(i), 1(j), Rule 3(a)	Billboard advertising using Santa Claus	**Upheld**—CYPA Principle 1, Rule 1(i), Rule 3a **Not upheld**—CYPA Rule 1(h), 1(j)
**5. McDonalds Restaurants NZ, Television (08/05/18) 18/107**	CYPA—Principle 1, Rule1(i), 1(j)	TV advertisement using children	**Not upheld**—CYPA Principle 1, Rule1(i), 1(j)
**6. Kinder Chocolate TV advertisement (12/06/18)**	CYPA—Principle 1, Rule 1(j), 1(k), Principle 2 Code for Advertising Food CFAF—Principle 1, Principle 2 Guidelines 2(a)2(b)	TV advertisement	**Not upheld**—CYPA Principle 1, Rule1(i), Rule1 (j) CFAF Principle 1, Principle 2, Guidelines 2(a)2(b)
**7. Kinder Chocolate TV online advertisement (30/10/18)**	CYPA—Principle 1, Principle 2 and Rules 1(i) and 2(f)	TV and online advertisement	**Not upheld**—Principle 1, Principle 2 and Rules 1(i) and 2(f)
**8. Cookie Time Limited Facebook and Instagram (9/04/19, 30/04/19)**	CYPA—Principle 1, Rule 1(e), 1(j) ASC—Principle 1, Rule 1(h)	Digital advertisement and billboard advertising (bus shelter)	**Upheld**—ASC Principle 1, Rule 1(h) **Not upheld** CYPA Principle 1, Rule 1(e),1(j)
**9. Arnott’s Tiny Teddy bus shelter and website (13/08/19)**	CYPA—Principle 1, Rule 1(e), Principle 2 ASC—Principle 1, Rule 1(h)	Bus shelter using school aged child Digital advertising (website) using school aged child	**Upheld**—Bus shelter—ASC Principle 1 and Rule 1(h) CYPA—bus shelter—Principle 1, Principle 2, Rule 1(e) **Not upheld** Website—CYPA and ASC
**Appeal Decision 1—Arnott’s Tiny Teddy bus shelter and website—appeal (9/12/19)**	Bus shelter—de novo	Bus shelter	**Not upheld**—CYPA—bus shelter—Principle 1, Principle 2, Rule 1(e)
**10. BurgerFuel Instagram and website advertisement (13/08/19)**	CYPA—Principle 1Rule 1(i), Rule 2(f) ASC—Principle 1, Rule 1(h), 2(g)	Digital advertising (Instagram and website)	**Settled** in part **Upheld** Instagram ASC Website—ASC Principle 1 Rule 1(h), 2(g) **Not upheld** Instagram—CYPA Website—CYPA
**11. Red Bull Digital Marketing Facebook, Instagram, Website (10/07/18)**	CYPA—Principle 1, Principle 2, Rule 1(h), 1(i), 1(j)	Digital advertising using famous person Sponsorship	**Not upheld** CYPA—Principle 1, Principle 2, Rule 1(h), 1(i), 1(j)
**12. Wilson Consumer Products Out of Home (15/11/18)**	CYPA—Principle 1, Rule 1(i) Principle 3, Rule 3(a)	Banners advertising confectionary at school aged sports event	**Settled**—as ad removed
**13. Hell Pizza, Digital Marketing (11/12/18)**	CYPA—Principle 1, Rule 1(i)	Digital advertising using children	**Not upheld** CYPA—Principle 1, Rule 1(i)
**14. KFC’s television advertisement (08/04/19)**	ASC—Principle 1, Rule 1(h), Principle 2, Rule 2(g) CYPA Code—Principle 1, Rule 1(h),1(i),1(j)	TV advertising using famous sports people	**Not upheld** ASC—Principle 1, Rule 1(h), Principle 2, Rule 2(g) CYPA Code—Principle 1, Rule 1(h),1(i),1(j)
**15. Griffins Food Company—Toffee pops (10/07/18)**	CYPA—Principle 1, Rule 1(c) Rule 1(i)	TV advertisement using famous sports people	**Not upheld** CYPA—Principle 1, Rule 1(c) Rule 1(i)
**16. Unilever Australasia (24/09/19)**	ASC—Principle 1 and Rule 1(h); CYPA—Principle 1; Rule 1(i) and Principle 2	Ice cream advertisement outside food store	**Upheld** ASC—Principle 1 and Rule 1(h) **Not upheld** CYPA—Principle 1; Rule 1(i) and Principle 2
**Appeal Decision 2 E Fowler—Unilever Australasia (25/11/19)**	ASC—Principle 1 and Rule 1(h); CYPA—Principle 1; Rule 1(i) and Principle 2	Ice cream advertisement outside food store	**Not upheld** ASC—Principle 1 and Rule 1(h); CYPA—Principle 1; Rule 1(i) and Principle 2
